# Diagnostic role of whole body bone scintigraphy in atypical skeletal tuberculosis resembling multiple metastases: a case report

**DOI:** 10.1186/1752-1947-3-141

**Published:** 2009-11-20

**Authors:** Majid Assadi, Iraj Nabipour, Mohammad Eftekhari, Abdolali Ebrahimi, Seyed-Reza Abotorab, Hooman Salimipour, Hamid Javadi, Katayon Vahdat, Reza Ghasemikhah, Mohsen Saghari

**Affiliations:** 1 Department of Nuclear Medicine, The Persian Gulf Biomedical Research Institute, Bushehr University of Medical Sciences, Bushehr, Iran; 2 Research Institute for Nuclear Medicine, Shariati Hospital, Tehran University of Medical Sciences, Tehran, Iran; 3 Department of Neurology, Faculty of Medicine, Shariati Hospital, Tehran University of Medical Sciences, Tehran, Iran; 4 Department of Nuclear Medicine, 5th Azar Hospital, Golsetan University of Medical Sciences, Gorgan, Iran

## Abstract

**Introduction:**

Osseous tuberculosis can be present with unifocal or multifocal bony involvement. Although multifocal involvement of the skeletal system in areas where tuberculosis is endemic is not a rare presentation, its exact prevalence is not well known. A case of atypical skeletal tuberculosis mimicking multiple secondary metastases on radiologic and scintigraphic imaging is presented to emphasize the contribution of bone scintigraphy in the assessment of osseous tuberculosis in typical and atypical presentations.

**Case presentation:**

A 73-year-old cachectic Asian man (Iranian) presented with a general feeling of being unwell and an acute loss of vision in his left eye accompanied by a severe headache. A Tc-99 m-methylene diphosphonate bone scan demonstrated multiple regions of intense activity in the appendicular and axial skeleton, suggesting metastatic involvement. Tumor markers (PSA, CA125, CA 19-9 and AFP) were within normal ranges. Based on clinical presentation and laboratory, radiological and scintigraphic findings, a presumptive diagnosis of tuberculosis was made. Quadruple antituberculous chemotherapy was consequently started and the patient later showed marked improvement.

**Conclusion:**

Scintigraphic bone scanning should be kept in mind when assessing bone pain in patients at a high risk of tuberculosis infection or reactivation. We present this unusual case of multifocal skeletal tuberculosis, and stress the related clinical and diagnostic points with the aim of stimulating a high index of suspicion that could facilitate early diagnosis and appropriate treatment.

## Introduction

Tuberculosis (TB) is still common in the developing world, so common that it must be considered in the differential diagnosis of substantial medical, surgical and gynaecological presentations [1].

The prevalence of osseous and extra-pulmonary TB is reported to be 1-5% [2,3] and 10-15% [3,4] of all TB infections, respectively. Osseous TB can be present with uni- or multifocal bony involvement. Although multifocal involvement of the skeletal system in areas where TB is endemic is not a rare presentation, its exact prevalence is still not well known. In some studies up to 7-13% of all osseous cases are multifocal [5,6]. Even though the scintigraphic pattern of this disease is recognized [2,5-8], some junior physicians might still be unfamiliar with this presentation.

A case of atypical skeletal TB mimicking multiple secondary metastases on radiologic and scintigraphic imaging is presented to emphasize the contribution of bone scintigraphy in the assessment of osseous TB in typical and atypical presentations.

## Case representation

A 73-year-old cachectic Asian man (Iranian) presented with a general feeling of being unwell and an acute loss of vision on his left eye accompanied by a severe headache. He did not have pain in any other parts of his body. He had a history of skin lesions on his buttocks, back and arms that were painless, dry and mobile. A malignant process that was mainly pancreatic in origin was thus suspected.

**Figure 1:**
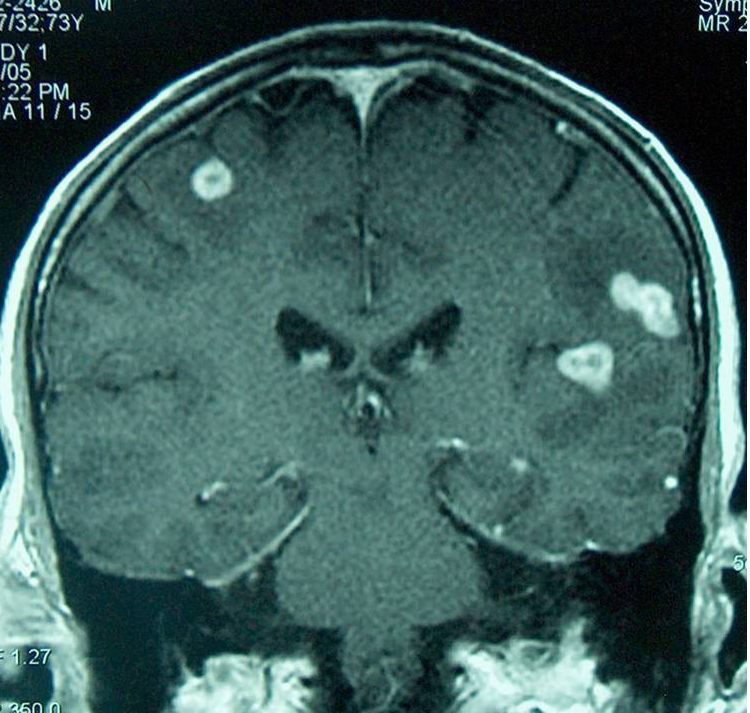
**Magnetic resonance image scan of the brain showing multiple brain masses with contrast enhancement and edema**.

Through a brain magnetic resonance image (MRI) scan on the patient, multiple brain masses with contrast enhancement and edema were detected (Figure 1). His chest computed tomography (CT) scan showed few cavities in the upper lobes of his lung. Results of his abdominal CT and ultrasonography scans were also normal. The patient underwent lumbar puncture, which was negative for TB in direct smear or polymerase chain reaction tests. The culture and cytology of the specimen also showed a negative result. Tumor markers such as PSA, CA125, CA 19-9, and AFP were all within normal ranges. Meanwhile, his elevated erythrocyte sedimentation rate (first hour was 70 mm/hr) and highly reactive C-reactive protein were of significance. Fine needle aspiration of the patient's skin lesions revealed evidence of bacterial infection but was negative for mycobacterium tuberculosis.

**Figure 2:**
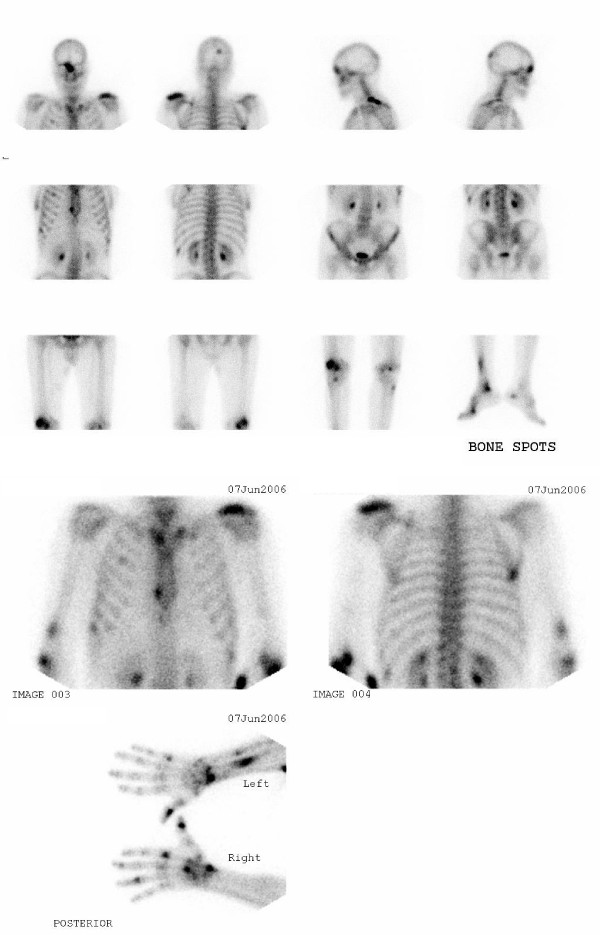
**Whole body bone scan revealed multiple foci of increased activities in the skull (right occipitoparietal and right orbitofrontal area), left clavicle, right lower sternal border, right sternoclavicular region, ribs, right distal humerus, left forearm, wrists, small bones of hands, lateral condyle of the right femur, right tibia, right ankle and the right first metatarsophalangeal joint**.

The PPD (purified protein derivative) skin test was positive and showed 20-mm indurations. A Tc-99 m-methylene diphosphonate bone scan demonstrated multiple regions of intense activity on the patient's appendicular and axial skeleton, including his skull (right occipitoparietal and right orbitofrontal areas), left clavicle, right lower sternal border, right sternoclavicular region, ribs, right distal humerus, left forearm, wrists, hands, lateral condyle of the right femur, right tibia, right ankle and right first metatarsophalangeal joint. All were suggestive of metastatic involvement (Figure 2).

Based on the patient's clinical presentation, laboratory, radiological and scinitigraphic findings, a presumptive diagnosis of TB was made and quadruple anti-TB chemotherapy (rifampin, isoniazid, pyrazinamide and ethambutol) was started. The patient later showed a marked improvement with weight gain and increased appetite after one month. Six months post-treatment, he was doing extremely well and had already returned to his normal daily life.

## Discussion

TB is a mycobacterial infection leading to morbidity and mortality of patients in many different regions of world. The main cause of this disease is *Mycobacterium tuberculosis*, while *M. bovis *and *M. africanum *can also produce this infection. Other mycobacterium species lead to infantile local adenopathy, which is usually without pulmonary or disseminated involvement especially in healthy subjects. The predisposing factors for TB include: recent TB contact, previous pulmonary TB, lower socioeconomic class, alcohol abuse, trauma, previous steroid therapy, diabetes mellitus, sarcoidosis, silicosis and immunodeficiency [9,10].

Although the prevalence of TB has declined steadily in Western Europe and North America, the global TB burden appears to be increasing, especially in the former Soviet Union, Eastern Europe and in Africa. HIV co-infection accounts for much of the recent increase in the global TB incidence [11].

The skeletal localization of TB is mostly seen in the dorsolumbar spine and diagnosis is often delayed. The presence of multiple TB sites can mimic secondary metastases, and biopsy remains the mainstay for final diagnosis. In 75% of cases, the lungs are the primary focus of the disease. Clinical manifestations are often mild and indolent. Therefore, early suspicion and diagnosis can prevent long-term morbidity and mortality.

Varying prevalence of skeletal TB has been reported. In the USA, skeletal TB comprises about 1-2% of all TB infections and 10% of extra-pulmonary infections [7]. In another study, skeletal TB was reported as 15% of all extra-pulmonary infections [6]. The commonly involved sites in adults are the skull, axial skeleton, the shoulders and the pelvic regions. In children, the metaphyseal regions of long bones are mostly involved and the most common site is the vertebral column, especially the lower thoracic and lumbar spine [8]. Skeletal TB lesions are usually solitary. Multifocal skeletal TB is defined as osteo-articular lesions that occur simultaneously at two or more locations. The prevalence of multifocal TB lesions differs in a variety of studies from between 7-10% [5] to 13% [6]. Multifocal skeletal TB lesions may mimic a malignant process both clinically and radiographically. On the other hand, osseous TB sometimes presents before the disease is diagnosed, as in this case. Skeletal scintigraphy is usually more sensitive than radiological imaging and detects more asymptomatic lesions. Multifocal TB and bony lesions are presented with multiple hot foci resembling skeletal metastases. In addition, degenerative changes, multiple fractures, myeloma, osteomalacia (pseudo-fractures), multifocal tumors (eosinophilic granuloma or lymphoma, histiocytosis X and fibrous dysplasia) and multiple osteomyelitis should be considered in differential diagnosis [6,12,13]. To prevent missed or delayed diagnosis, it is necessary to consider skeletal TB in differential diagnosis especially in patients in whom the origin of the disease is unknown.

Since TB can be present in multiple sites, especially in patients from areas where TB is endemic, it is essential to avoid a delay in diagnosis.

Recently, more cases of this kind of presentation have been seen in immunodeficient patients, especially in heroin users and HIV-positive people.

Bone scintigram is not generally required in the investigation of TB. The most common reason for the ordering the test is bone pain, which may precede the diagnosis of TB. In some cases, a whole-body bone scan also allows the identification of an asymptomatic lesion, providing thus a site for biopsy and early diagnosis.

Our case revealed atypical disseminated lesions located in the axial and appendicular regions. In this case there were multiple lesions in the small bones of the hands and feet, as well as in the wrists, distal forearm and legs. This pattern of lesion distribution is less likely to be related to a skeletal tumor (primary or secondary), so other possibilities like TB should be kept in mind, especially in regions where the disease is endemic.

## Conclusion

In areas where TB is endemic, multifocal skeletal TB is not rare; therefore, in analysing a bone scan that shows multiple lesions, multifocal skeletal TB should be considered in the differential diagnosis especially when the origin of the disease is unknown.

In addition, a skeletal scintigraphy can show the extent and distribution of osseous lesions in cases of suspected skeletal TB. In the early stages, when radiological imaging such X-ray, MRI or CT is normal, a whole body bone scan may help detect lesions and provide a site for biopsy diagnosis. Finally, scintigraphic bone scanning should be kept in mind when assessing bone pain in patients who are at high risk for TB infection or reactivation.

## Abbreviations

CT: computed tomography; MRI: magnetic resonance imaging; TB: tuberculosis.

## Consent

Written informed consent was obtained from the patient for publication of this case report and any accompanying images. A copy of the written consent is available for review by the Editor-in-Chief of this journal.

## Competing interests

The authors declare that they have no competing interests.

## Authors' contributions

MA participated in the design, coordination and drafting the manuscript, and also interpreted the radiological figures. ME, SRA, HS, HJ, KV and MS supervised the acquisition and interpretation of the radiological images. IN, AE and RG revised the article for important intellectual content and helped draft the manuscript. All authors read and approved the final manuscript.
